# Epidemic Varicella Zoster Virus among University Students, India

**DOI:** 10.3201/eid2402.170659

**Published:** 2018-02

**Authors:** Josh Meyers, Muthunarayanan Logaraj, Balaji Ramraj, Padmanesan Narasimhan, C. Raina MacIntyre

**Affiliations:** University of New South Wales, Sydney, New South Wales, Australia (J. Meyers, P. Narasimhan, C.R. MacIntyre);; Sri Ramaswamy Memorial University, Chennai, India (M. Logaraj, B. Ramraj);; Arizona State University, Phoenix, Arizona, USA (C.R MacIntyre)

**Keywords:** Varicella, zoster, viruses, chickenpox, herpesvirus, outbreak investigation, Chennai, India, Uttar Pradesh, Odisha, university, students, adults, dormitory, hostel, tropical, temperate, climate, immunization, vaccine, maculopapulovesicular rash, lesion, shingles, isolation

## Abstract

We investigated a yearlong varicella zoster virus outbreak in a highly susceptible young adult population at a large university in India. Outbreaks of varicella infection among adults are not well described in the literature. Infection control measures and vaccination policy for this age group and setting are needed.

Infection with varicella zoster virus (VZV; also known as chickenpox virus), a human herpesvirus, occurs worldwide, and most persons living in temperate regions become immune by contracting varicella illness or receiving vaccinations by early adulthood (*1*,*2*). However, in tropical climates, as little as half the population is exposed to VZV by adulthood (*1*). The extent of disease and severity in adults in tropical climates is also greater than for temperate climates (*1*,*3*). In India, *>*30% of persons >15 years of age are susceptible (*4*). Routine infant vaccination has substantially reduced transmission of wild-type varicella (*3*); however, VZV vaccine is not a part of the Indian Universal Immunisation Program (*5*). We describe an outbreak of varicella in 2016 at a large private university in Chennai, India.

## The Study

 The university at which the outbreak occurred has previously experienced recurrent varicella epidemics that have not been formally investigated. The university had 6,000 staff and 40,000 students (domestic and international) in 28 schools and colleges. The campus includes a 1,200-bed hospital, and residents are housed in >25 dormitory-style hostel blocks, most of which, except block 1, have single-sex occupancy.

We identified all cases among students or staff who received a varicella diagnosis at the university hospital during February 2016–January 2017 by using the hospital’s notifiable disease register. We selected controls by using systematic stratified sampling by hostel block at a ratio of 1:1 and matched according to age, sex, and hostel; we excluded those who self-reported a history of varicella illness.

Hospital clinicians used a clinical case definition to diagnose varicella: an illness with acute onset of diffuse maculopapular vesicular rash without another cause (*6*). No laboratory tests were done. Severity of disease was defined by the number of lesions (*7*).

We distributed questionnaires to eligible students and staff and sought written informed consent for participation in the study. Information gathered included demographics; disease characteristics; and risk factors such as region of birth, contact with varicella, living quarters, and study program. Vaccination status was determined by participants’ recall and defined as receipt of any varicella-containing vaccine. We calculated denominators for dormitories from occupied bed data at the start of the 2016–2017 semester and determined rates by state of residence by using the number of enrolled students from each state in India according to university dormitory and admissions records.

We used Microsoft Excel (Microsoft, Redmond, WA, USA) and IBM SPSS 21 (IBM, Armonk, NY, USA) to analyze the data. We used descriptive statistics calculated by using the Pearson χ^2^ test for comparison of proportions and the Mann-Whitney U test for comparison of nonparametric variables. A p value of <0.05 was considered statistically significant. Ethics approval was obtained from Sri Ramaswami Memorial University Ethics Committee, Chennai, India (ethics clearance no. 1160/IEC/2017).

**Figure 1:**
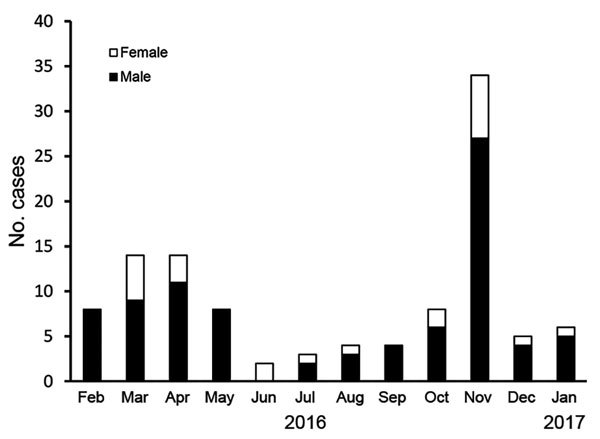
Cases of varicella in a private university in Chennai, India, February 2016–January 2017, by sex and month of admission to hospital. Academic examinations occur during April–May and November–December; semester holidays occurred during June and December 2016.

During February 2016–January 2017, a total of 110 cases of varicella were diagnosed at the university hospital. Of these, 87 (79%) case-patients were male, 23 (21%) were female, and 100 (91%) were residents in the dormitory blocks of a single college. A peak of 34 cases was reported in November 2016 (Figure 1).

**Figure 2:**
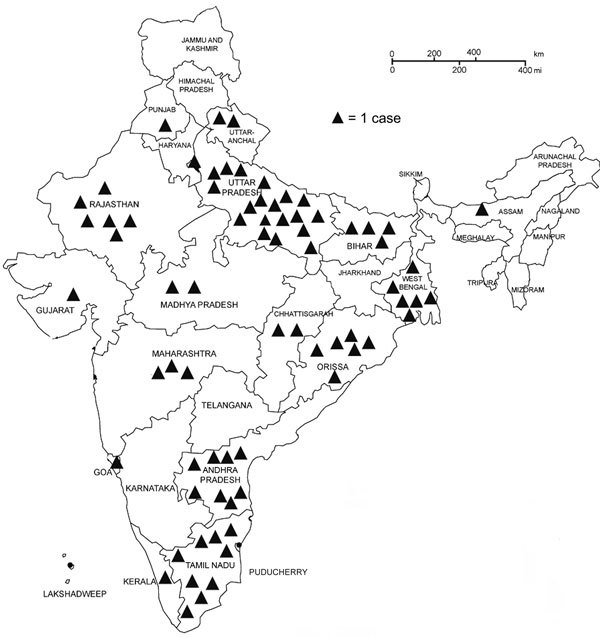
States of birth for 73 varicella case-patients at a private university in Chennai, India, February 2016–January 2017. Each triangle indicates 1 case. Incidence of varicella (per 1,000 students) by state of birth: Odisha, 16.0; Uttar Pradesh, 9.8; Andhra Pradesh, 2.1; Tamil Nadu, 1.8; other, 3.3.

Among the 110 case-patients, 77 completed the questionnaire and were included in further analysis (Table 1). Students born in Odisha had the highest incidence (16 cases/1,000 students); those from Uttar Pradesh had the second-highest incidence (9.8 cases/1,000 students) (Figure 2).

**Table 1:** Demographics of participants in study of varicella outbreak at a private university in Chennai, India, February 2016–January 2017*

Characteristic	Case patients, n = 77	Controls, n = 77†	p value
Sex, %			
M	63 (81.8)	63 (81.8)	NS—matched variable
F	14 (18.2)	14 (18.2)	NS—matched variable
Age, y			
Median	18	18	NS—matched variable
Range	17–40	17–52	NS—matched variable
State of birth, % (SE)			0.01
Uttar Pradesh	19 (24.7–4.9)	6 (7.8–3.1)	
Tamil Nadu	9 (11.7–3.7)	14 (18.2–4.4)	
Andhra Pradesh	8 (10.4–3.5)	9 (11.7–3.7)	
Odisha	6 (7.8–3.1)	1 (1.3–1.3)	
Other/NA	35 (45.5–5.7)	47 (61.0–5.6)	
Degree program,‡ % (SE)			0.95
Bachelor of Technology	70 (90.9–3.3)	70 (90.9–3.3)	
Bachelor of Medicine, Bachelor of Surgery, Doctor of Medicine and Research	2 (2.6–1.8)	3 (3.9–2.2)	
Master of Technology	1 (1.3–1.3)	1 (1.3–1.3)	
NA	4 (5.2–2.5)	3 (3.8–2.2)	
Student year or staff position, % (SE)			0.82
1	63 (81.8–4.4)	65 (84.4–4.1)	
2	8 (10.4–3.5)	7 (9.1–3.3)	
3	1 (1.3–1.3)	2 (2.5–1.8)	
Staff	5 (6.5–2.8)	3 (3.8–2.2)	
Campus category, %			NS—matched variable
Resident	72 (93.5)	70 (90.9)	
Day student	1 (1.3)	4 (5.2)	
Staff	4 (5.2)	3 (3.8)	
Self-reported vaccination for varicella, % (SE)			0.10
Yes	26 (33.8–5.4)	36 (46.8–5.7)	
No/not sure	51 (66.2–5.4)	41 (53.2–5.7)	
Known exposure to varicella during study period, % (SE)			0.03
Yes	26 (33.8–5.4)	14 (18.2–4.4)	
No known exposure	51 (66.2–5.4)	63 (81.8–4.4)	

*NS, not stated; NA, not available. †Controls were matched by age, sex, and place of residence (hostel residents were matched by hostel block). ‡Degree program was suspected as potentially contributing to transmission of the disease by close contact during classes.

The infection predominantly affected resident students; 72 study participants (94%) resided in a dormitory, whereas the other 6% lived in private housing (Table 1). The attack rate in dormitories was 14 cases/1,000 residents; the rate was lower in women’s dormitories than in men’s (Technical Appendix Table). Dormitory block 5 had the highest attack rate (23 cases/1,000 residents).

Of the 77 case-patients who completed the questionnaire, 25 (32.5%) reported attending class during their illness; 67 (87%) were isolated at some point, either at home or in the hospital. A rash was reported by all case-patients, fever by 59 (77%), cough by 24 (31%), and malaise by 34 (44%). The number of days absent from work or study was lower for vaccinated than nonvaccinated groups (6.2 days vs. 8.0 days; p = 0.046). The number of days from onset of symptoms to hospitalization ranged 0–11 days (median 2 days) (Table 2).

**Table 2:** Characteristics of 77 patients with varicella in study of varicella outbreak at a private university in Chennai, India, February 2016–January 2017

Characteristic	Value
Symptoms, no. (%) patients	
Fever	59 (76.6)
Rash	77 (100.0)
Cough	24 (31.2)
Malaise	34 (44.2)
Illness duration, no. (%) patients	
<1 week	11 (14.3)
1–2 weeks	54 (70.1)
>2 weeks	12 (15.6)
No. days off work or study	
Median	7
Range	0–18
Attend class while ill, no. (%) patients	
Yes	25 (32.5)
No	52 (67.5)
Kept in isolation, no. (%) patients*	
Yes	67 (87.0)
No	10 (13.0)
No. days from onset of symptoms to hospital admission†	
Median	2
Range	0–11
No. case-patients who responded “not sure”	27

*Isolation indicates a patient spent time in an isolation room in the hospital or was in isolation at home at any point during the illness. †Those who responded “not sure” were excluded from calculation.

## Conclusions

Varicella epidemics among adults are not described well in the literature. This study describes a year-long propagated epidemic in a university in India with a background of past epidemics. A large student body with low immunization rates living in dormitories provides fertile ground for transmission of varicella and highlights a potential role for vaccination in young adults in India. Although studies in India and Thailand show that community transmission and household crowding predispose to varicella epidemics (*8*), transmission in a tertiary education setting has not been described. A serologic study of undergraduate students in Sri Lanka further suggests high susceptibility in this cohort (*9*).

With a highly susceptible young adult population living in a densely populated campus setting, adequate infection control strategies must be practiced (*6*). Approximately 33% of case-patients attended class while ill, and 13% were not kept in isolation postdiagnosis. Furthermore, a median of 2 days from onset of symptoms passed before hospitalization, increasing potential for transmission of infection before isolation. In addition to students being in close contact on campus, adherence to infection control procedures was poor, possibly leading to the increased transmission of VZV, as described by Greenaway et al. (*8*).

Vaccination can control epidemics but was not used in this setting (*4*,*6*,*10*). Estimates of vaccination of adults in India are ≈20% (*3*), lower than the 34% self-reported figure in this study, which suggests recall bias by the patients. A serologic study and validation of vaccination history of participants would assist in examining this factor in more detail.

The usual seasonal peak of varicella infections in southern India is January–April (*6*). The peak incidence during this study was in November and April; the larger November peak represented a marked variation from the usual seasonal pattern. From a policy perspective, it would be necessary to ascertain the frequency and magnitude of university epidemics, geographic hot spots, and the overall burden of disease in India. Immunization in settings such as universities would reduce transmission and is an essential intervention during an acute epidemic (*6*,*11*).

Most students affected were teenage boys in their first year of study. The analysis by state of birth showed high incidence of varicella for students born in both Uttar Pradesh and Odisha. Different climate and susceptibility by states should be investigated further to inform disease control efforts in India.

This study has several limitations. We had no confirmatory laboratory data; however, the clinical syndrome of varicella is fairly unique. The study is subject to recall bias on vaccination history, previous exposure, disease characteristics, and respondent bias. Furthermore, only case-patients whose medical records included a positive diagnosis were included in the study. Active case finding was not done, so the true incidence is likely higher. We were unable to ascertain the index case for the epidemic.

This study describes a substantially circulated, yearlong adult varicella epidemic at a large university in India. Dormitory students were most affected, but day students and staff were also affected. A substantial proportion of case-patients attended classes while ill, highlighting the need for active case finding and isolation during epidemics. A larger study is required to inform vaccination policy and disease control. Appropriate infection prevention and control strategies as well as use of vaccination during outbreaks should be considered.

Technical Appendix Attack rate of varicella by dormitory and total number of cases recorded at a private university in Chennai, India, during February 2016–January 2017.
