# Supplementary material for: Epidemic Varicella Zoster Virus among University Students, India
Source: Emerg Infect Dis. 2018 Feb;24(2):366–9. doi: 10.3201/eid2402.170659 (PMC5782884; doi:10.3201/eid2402.170659)
Supplement: Technical Appendix — Attack rate of varicella by dormitory and total number of cases recorded at a private university in Chennai, India, during February 2016–January 2017. [file 17-0659-Techapp-s1.pdf]

Article DOI: https://doi.org/10.3201/eid2402/eid2402170659

# Epidemic Varicella Zoster Virus among University Students, India

## Technical Appendix

**Technical Appendix Table.** Attack rate of varicella among college students by dormitory and total number of cases recorded at the University Hospital over 12 months*

| Dormitory | Cases | Occupied beds | Rate per 1,000 university dormitory residents |
|---|---|---|---|
| Block 1 (male and female) | 3 | 813 | 4 |
| Block 2 (male only) | 7 | 966 | 7 |
| Block 3 (male only) | 13 | 938 | 14 |
| Block 4 (male only) | 17 | 963 | 18 |
| Block 5 (male only) | 23 | 976 | 23 |
| Block 6 (male only) | 2 | 544 | 4 |
| Block 7 (female only) | 1 | 549 | 2 |
| Block 8 (female only) | 3 | 539 | 5.5 |
| Block 9 (female only) | 4 | 683 | 6 |
| Total | 100 | 6,971 | 14 |

*Includes all recorded cases from university campus hostels, including those who did not complete the study questionnaire.
